# Anti-Angiogenic RNAi-Based Treatment of Endometriosis in a Rat Model Using CXCR4-Targeted Peptide Nanoparticles

**DOI:** 10.3390/ijms262110582

**Published:** 2025-10-30

**Authors:** Anna Egorova, Svetlana Freund, Iuliia Krylova, Anastasia Kislova, Anton Kiselev

**Affiliations:** 1 Laboratory of Molecular Genetics and Gene Therapy, D.O. Ott Research Institute of Obstetrics, Gynecology and Reproductology, Mendeleevskaya Line 3, 199034 Saint-Petersburg, Russia; egorova_anna@yahoo.com (A.E.);; 2 Department of Pathology, Pavlov First Saint-Petersburg State Medical University, L’va Tolstogo Street 6-8, 197022 Saint-Petersburg, Russia

**Keywords:** endometriosis, anti-angiogenic therapy, siRNA delivery, peptide-based carriers, gene therapy, VEGFA, CXCR4, chemokine-derived peptide

## Abstract

Endometriosis is a common gynecological condition that affects fertility in many women of reproductive age worldwide. This multifaceted disease exhibits a pathogenesis characterized by hormonal and immune system dysregulations, alongside increased angiogenic activity within the peritoneum. The aberrant proliferation of endometrial tissue outside the uterus is associated with vascularization in ectopic endometriotic lesions. Consequently, RNA interference (RNAi)-based angiogenic therapies targeting the *VEGFA* gene present a promising strategy for the treatment of endometriosis. To ensure the efficacy of RNAi-based therapy, it is critical to develop carriers capable of precisely delivering small interfering RNA (siRNA) to target cells. Additionally, the instability of polyplexes in vivo must be regarded as a pivotal aspect influencing the success of non-viral delivery. In this study, we introduce ternary polyplexes comprising siRNA and a carrier derived from an arginine–histidine-rich peptide, which is further coated with a glutamate–histidine-rich polymer modified using an SDF-1 chemokine-derived ligand for targeting CXCR4-expressing cells. The physicochemical characteristics of the siRNA-polyplexes, along with cellular toxicity and *GFP* gene silencing efficacy, were assessed in vitro. The anti-angiogenic potential of anti-VEGFA siRNA-polyplexes was evaluated by measuring the size of endometrial lesions, conducting immunohistochemical staining, and analyzing *VEGFA* gene expression. For in vivo experiment, a rat model of endometriosis induced by subcutaneous auto-transplantation of uterine tissue was utilized. A significant reduction in the growth of endometriotic implants and silencing of *VEGFA* gene expression was observed when compared to the saline-treated control group. The results of this study strongly suggest that the developed ternary polyplexes have significant potential as an efficient tool for the development of anti-angiogenic RNAi-based therapies for endometriosis.

## 1. Introduction

Endometriosis (EM) is a common gynecological condition characterized by the abnormal growth of tissue that resembles the endometrium outside of the uterine cavity and is one of the leading causes of female infertility [1]. It is estimated that approximately 5–10% of women in reproductive age experience this condition, which corresponds to approximately 190 million women globally [2]. The most widely accepted theory of EM development is the implantation theory, which suggests that endometrial tissue can enter the abdominal cavity via the fallopian tube during menstruation and form ectopic foci [2]. Other factors, such as cell viability and growth, reduced immune response, and angiogenesis, are also significant in the development of the disease. These factors, when combined, lead to a decrease in the elimination of ectopic endometrial cells [3]. This is followed by their adhesion and proliferation, leading to the development of endometriotic lesions [4].

Currently, there are several established approaches to treating this condition. Hormone therapy results in suppression of ovulation and reduction in estrogen levels to postmenopausal values [5]. However, this treatment is associated with a number of adverse effects, as well as recurrence after discontinuation [6]. Surgical intervention is also an option, but it often only addresses symptoms and does not restore reproductive function [7]. Therefore, the development of efficient alternate therapy techniques that can restore reproductive capacity in women with EM without adverse effects is a significant and promising area of research.

RNAi-based therapies have the potential to be an alternative treatment for EM, as they are a rapidly developing approach to treating various diseases [8]. Therapeutic siRNAs can be effectively encapsulated and delivered using different non-viral vectors, e.g., lipids, polymers, peptides, inorganic particles, etc. [9]. Several previous experimental studies on EM models have confirmed the feasibility of this approach [10,11,12].

In case of EM an anti-angiogenic RNAi-based therapy can be suggested due to the fact that the growth and survival of the endometrial lesions is highly dependent on development of new blood vessels that supply the heterotopias with oxygen and nutrients [4]. The main pro-angiogenic factor in the endometrium is vascular endothelial growth factor (VEGFA). This is a glycoprotein that promotes the proliferation and migration of endometrial cells, as well as increases vascular permeability [13]. In the normal menstrual cycle, VEGFA levels change cyclically and reach their peak during the secretory phase. The expression of this factor is usually increased in response to estrogen and progesterone [14,15]. In women with EM, there is an increase in VEGFA levels in peritoneal fluid during the proliferative phase of the menstrual cycle [15]. Increased levels of VEGFA have been observed in both peritoneal fluid and ectopic lesions in patients with the disease, compared to control groups. These increased levels contribute to significant vascularization of endometrioid lesions [16].

It has been shown that systemic administration of angiostatic compounds can effectively suppress angiogenesis and growth of endometrial lesions in EM animal models, however often it results in undesirable inhibition of eutopic endometrium growth and impairment of fertility [17]. These results highlight importance of active drug targeting, e.g., by application of receptor-mediated delivery [18]. It has been demonstrated that under the influence of VEGFA and other factors, the migration and differentiation of endothelial progenitor cells (EPC) occurs, leading to the formation of new blood vessels [19]. This process is facilitated by the action of SDF-1 (CXCL12), a stem cell migration factor, and its receptor, CXCR4. The interaction between these molecules induces the attraction of EPC to the site of EM, where it contributes to the development and progression of the disease [20,21]. In EM, there is an increase in both the level of SDF-1 and CXCR4. It has been demonstrated that the levels of the CXCR4 protein are significantly higher in endometriotic foci compared with the endometrium in the control group [22]. Modeling EM in rats has demonstrated an increase in expression of the CXCR4 gene [23]. Women with EM have higher levels of CXCR4 in ovarian endometriotic tissue and in the glandular epithelial cells of both ectopic and eutopic endometrial tissue compared to the control group [24]. Additionally, in ovarian endometriosis, expression of the CXCL12 gene is significantly higher in ectopic endometriotic lesions and cancer compared to normal ovarian tissue [24]. It should be noted that the process of the receptor-ligand binding is well understood, and specific peptide sequences have been developed for targeting CXCR4 [25,26,27]. Therefore, CXCR4 could be selected as a target for the specific delivery of EM lesions.

Previously, we developed peptide nanoparticles (NP) that were modified with a CXCR4 ligand and carried anti-VEGFA siRNA. In vitro studies using an endothelial hybridoma and in vivo studies using a rat model demonstrated an efficient downregulation of the target gene expression, subsequent anti-angiogenic effects and a significant reduction in the volume of experimental EM lesions [11,28,29].

Although lipo- and polyplexes are widely studied for their potential use in therapeutic RNA delivery, their high positive charge density raises concerns about toxicity and limited diffusion in the extracellular space of the target tissue, which may restrict their clinical applications [30,31,32]. A high cationic charge can lead to adverse effects due to damage to the cell membrane, off-target distribution, or elimination through nonspecific protein absorption in the bloodstream [33]. The charge of cationic supramolecular complexes can be neutralized to improve their biodistribution in vivo [34,35]. One potential approach is to modify these complexes with polyethylene glycol (PEG) molecules [36]. While PEGylation can significantly extend the circulation half-life of a delivery system, several studies have demonstrated a loss of long-term circulation properties in PEGylated complexes after repeated intravenous administration due to an enhanced blood clearance process [37,38].

An alternative approach is to employ anionic polymers in order to minimize undesired electrostatic interactions and consequently reduce toxicity. It has been previously hypothesized that the penetration through tissue barriers could be enhanced by decreasing the surface charge of the polyplex [39,40,41,42]. More recently, our research and that of others has demonstrated that an anionic coating is able to shield the positive surface charge of a polyplex, thereby reducing non-specific interactions and improving serum stability [32,41,43]. A serum-resistant peptide-based DNA delivery systems developed using this approach have proved successful in transfecting uterine fibroids and muscle tissues [32,41,44].

We describe here the development of an siRNA delivery system that includes bioreducible cystine-crosslinked polyglutamic acid as an anionic coating component. The physicochemical properties of peptide NPs were characterized, and their toxicity and transfection capabilities were explored in CXCR4-expressing cancer cells. The therapeutic potential of the siRNA delivery system was evaluated in a surgically induced subcutaneous EM rat model treated by anti-VEGFA siRNA.

## 2. Results

### 2.1. Design of Carriers

In this study, we have characterized a novel siRNA delivery system based on a polycondensed cross-linking peptides. The system consists of two components: a cationic R6p module that binds siRNA, which has been previously shown to be an efficient carrier for nucleic acids (NA), and an anionic CDP-E6pH coating module, which is modified with the N-terminal fragment of the SDF-1 chemokine (CDP—chemokine derived peptide) [28,45]. This fragment has been shown to act as a specific ligand for CXCR4 [26].

The whole delivery system, designated as R6p/CDP-E6Hp, is designed using a modular approach to overcome the extra- and intracellular barriers of NA transport into cells [46]. The carriers’ design is shown in Table 1.

### 2.2. EEvaluation of Polyplexes Relaxation by Anionic Coating

The main function of the anionic coating in the formulation of the developed polyplexes is to maintain stability when in contact with extracellular components, primarily negatively charged proteins and glycosaminoglycans (GAG), which can cause dissociation of NA from the carrier, reducing transfection efficiency [47]. However, the presence of an anionic polymer can destabilize a polyplex due to electrostatic repulsion, and this fact necessitates careful optimization of the formulation. Additionally, modification of the polyplexes with the CDP ligand may also affect their stability, so a ligand-free coating was used as a control to evaluate this.

**Figure 1:**
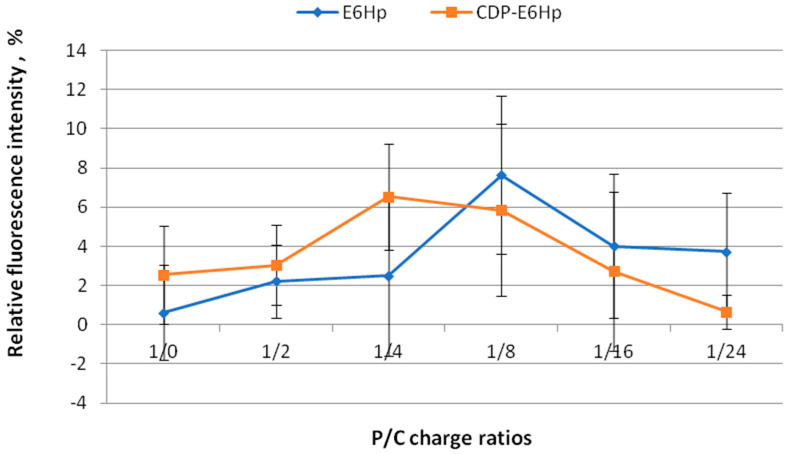
The relative fluorescence intensity of SybrGreen when binding to siRNA at different Phosphate/Carboxyl (P/C) charge ratios of siRNA/R6p complexes (siRNA/R6p ratio of 1/16) and anionic coating—E6Hp (blue) or CDP-E6Hp (orange). Values are the mean ± SD of *n* = 9 individual samples from three independent experiments.

The stability of siRNA/R6p complexes with anionic coating was assessed using SybrGreen exclusion assay. The siRNA/R6p complexes were prepared at a charge ratio of 1:16 to ensure dense packaging of siRNA [28]. These complexes were then coated with negatively charged polymers CDP-E6Hp or E6Hp at different P/N/C (Phosphate/Nitrogen/Carboxyl) ratios of 1:16:2, 1:16:4, 1:16:8, 1:16:16, and 1:16:24 to form final ternary complexes, which were used to determine the degree of siRNA binding. The fluorescence of naked siRNA with the intercalating dye was taken as 100% for comparison (Figure 1).

Based on the data presented in Figure 1, it can be concluded that the R6p carrier at a charge ratio of 1:16, in combination with various amounts of E6Hp and ligand-modified E6Hp coatings, successfully binds siRNA molecules. The relative fluorescence intensity of SybrGreen bound to the siRNA does not exceed 8%, and there is no difference between the control and ligand-modified polyplexes. This indicates that all the formulations tested are stable, and the interaction between cationic and anionic components does not lead to the dissociation of siRNA from the polyplex. These findings support our previous research, which showed that modification of anionic coating with a peptide ligand does not affect the ability of carriers to form complexes with DNA [44].

### 2.3. Size and Zeta-Potential of Ternary Polyplexes

The physicochemical parameters of the polyplexes used in this study were characterized using microelectrophoresis to determine zeta-potentials and dynamic light scattering to measure particle sizes. Ternary complexes formed at P/N/C ratios 1:16:4, 1:16:8, 1:16:16, and 1:16:24 were tested in the experiments.

**Figure 2:**
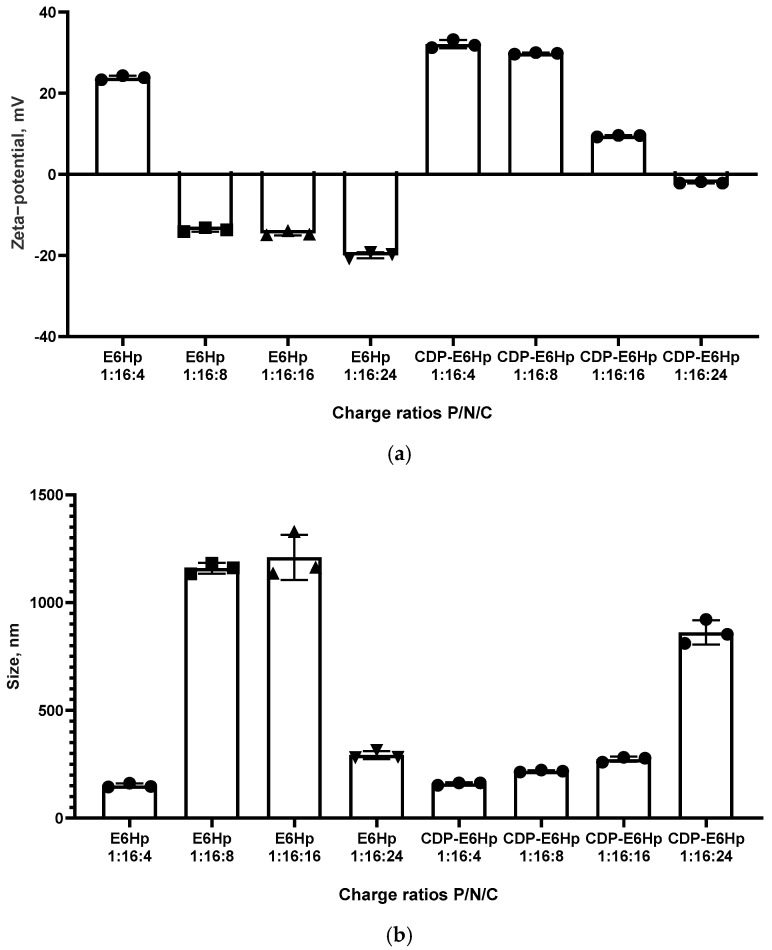
Zeta-potential (**a**) and size (**b**) of ternary polyplexes at different Phosphate/Nitrogen/Carboxyl (P/N/C) charge ratios of siRNA/R6p complexes (siRNA/R6p ratio of 1:16) with an anionic coating. Values are the mean ± SD of n = 3 individual samples from three independent experiments.

Previously, we demonstrated that siRNA/R6p polyplexes at a P/N ratio of 1:16 exhibited a strong positive charge, with a zeta potential of 33.2 mV, due to the cationic nature of the R6p carrier [48]. In this study, an anionic coating was added to neutralize the positive charge. According to the data presented in Figure 2a, a clear difference is observed between the zeta-potentials of E6Hp-coated and CDP-E6Hp-coated complexes of siRNA with the cationic R6p carrier at low P/N/C ratios (1:16:4). The addition of E6Hp coating only slightly neutralizes the positive charge of the siRNA/R6p polyplex at this ratio, resulting in a zeta potential value of 24.3 mV. However, the addition of CDP-E6Hp at this same charge ratio did not lead to a decrease in zeta potential.

Similarly, a sharp decrease in zeta-potential was observed only for siRNA/R6p/E6Hp formulations at P/C ratios ranging from 1:8 to 1:24, with zeta-potential values ranging from −13.7 to −19.3 mV. In contrast, siRNA/R6p/CDP-E6Hp polyplexes at the same charge ratios exhibited zeta-potential values ranging from 29.8 mV to −1.86 mV. This clearly indicates that the CDP-ligand modification of the polyplexes results in a lower degree of charge neutralization.

It has been established that zeta-potential values are closely associated with the stability of nanoparticles [49]. Systems that have surface charge values tending towards neutrality are more likely to aggregate [50]. One potential disadvantage is that polyplexes can become unstable and aggregation of particles may occur. However, a significant benefit is that this approach avoids interaction with proteins in the bloodstream in vivo [33]. In this instance, zeta-potential values for both polyplex types were neutral or slightly negative, consistent with the tendency towards neutralization of charges of all components following their interaction.

Size is considered an important physicochemical parameter of polyplexes, as it relates to their ability to penetrate cells [51]. We have previously shown that siRNA/R6p polyplexes formed with a P/N ratio of 1:16 and without an anionic component have an approximate size of 150 nm [48]. In this study, complexes of siRNA/R6p with the same charge ratio coated with E6Hp and CDP-E6Hp polypeptides exhibited a larger size in most formulations, ranging from approximately 151 nm to 1210 nm. It was expected that, due to the relatively high and positive zeta-potential values of the ternary polyplexes formed at a charge ratio of 1:16:4, they would be compact, as shown in Figure 2b. An aggregation was observed for three formulations, namely, for siRNA/R6p/E6Hp polyplexes formed at a charge ratio of 1:16:8 and 1:16:16, and for siRNA/R6p/CDP-E6Hp polyplexes at a charge ratio of 1:16:24 (Figure 2b). It should be noted that, in the case of E6Hp-coated polyplexes, aggregation occurred at zeta-potential values around −13 mV, whereas their CDP-E6Hp-coated analogues, at a charge ratio of 1:16:16 formed more compact polyplexes with a weak positive charge of 9 mV. Therefore, a clear distinction can be made between the polyplexes coated with E6Hp and those coated with CDP-E6Hp. The observed shift in zeta-potential after coating with the anionic polymer confirms the successful functionalization of the polyplexes.

### 2.4. Evaluation of the Cytotoxic Effects of Ternary Polyplexes

The MDA-MB-231 cell line was chosen for cellular experiments due to its expression of CXCR4 on the cell surface [52]. Additionally, it should be noted that this specific cell line, which stably expresses eGFP reporter gene, was used to investigate the efficacy of RNAi-based gene silencing with ternary polyplexes.

**Figure 3:**
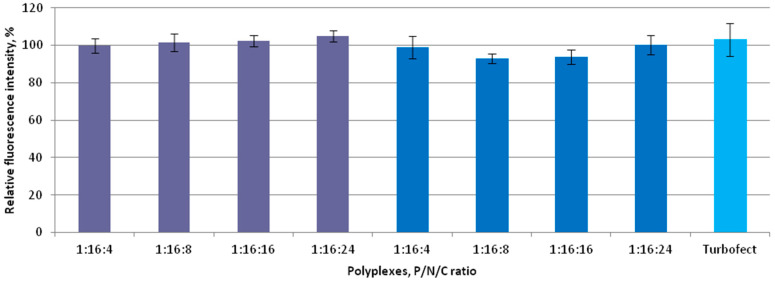
Cytotoxicity evaluation of the ternary polyplexes in MDA-MB-231 cells at different P/N/C ratios of siRNA/R6p complexes (siRNA/R6p ratio of 1/16) and anionic coating—E6Hp (violet) or CDP-E6Hp (blue). Values are the mean ± S.D. of n = 9 individual samples from three independent experiments.

Due to the pivotal role of cytotoxicity in evaluating the suitability of the investigated carrier systems for siRNA delivery, the polyplexes were tested for potential cytotoxic effects in vitro. The assessment was performed at P/N/C ratios of 1:16:4, 1:16:8, 1:16:16, and 1:16:24, with siRNA/Turbofect complexes serving as a positive control. The results shown in Figure 3 indicate that no significant differences in cytotoxicity were observed among the coated polyplexes across all tested charge ratios in MDA-MB-231 cells. Due to the fact that one of the major factors influencing the level of cytotoxicity caused by non-viral nanoparticles is their high positive charge, we anticipated a significant improvement in this parameter through charge neutralization [53]. Previously, we demonstrated that siRNA/R6p polyplexes with a charge ratio of 1:16 exhibited high cytotoxicity toward MDA-MB-231 and endothelial cells [48]. In this study, we show a significant reduction in the cytotoxic effects of ternary polyplexes. These findings are consistent with previous research on other types of non-viral delivery systems, which have shown that partial neutralization of positive charge can enhance the delivery of DNA and siRNAs [32,41,53,54,55].

### 2.5. Evaluation of the Trasnsfection Properties of Ternary Polyplexes In Vitro

**Figure 4:**
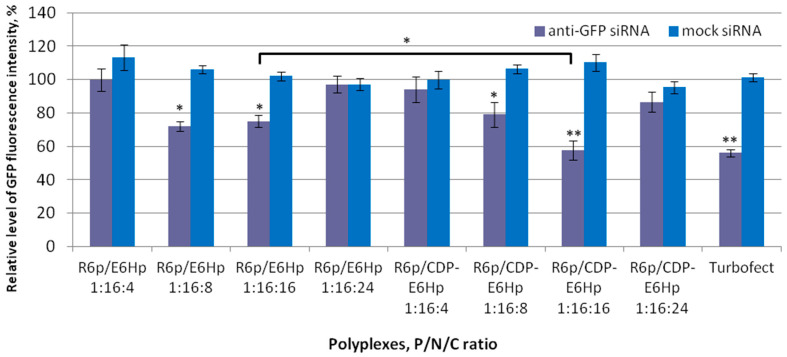
Silencing of GFP gene expression after treatment of MDA-MB-231 cells with the ternary polyplexes. *—*p* < 0.05, **—*p* < 0.01 when compared with negative control. The data are shown as the mean ± S.D. of n = 9 individual samples from three independent experiments; statistical significance was assessed by ordinary one-way ANOVA.

Transfection efficiency of the developed polyplexes was determined by gene silencing in GFP-expressing MDA-MB-231 cells by applying specific siRNA. Level of GFP fluorescence was assessed by spectrofluorimetry and the level in intact cells was taken as 100% for comparison (Figure 4). Specificity of RNA-based gene expression silencing was confirmed by using mock siRNA as a negative control, whereas siRNA/Turbofect complexes were used as a positive control. Additionally, representative images of GFP-expressing MDA-MB-231 cells were recorded using fluorescent microscopy after RNAi-mediated gene expression silencing (Figure S1).

The results of gene expression silencing are illustrated in Figure 4. The data indicate that no silencing effect was detected when polyplexes were formed using a mock siRNA, thereby corroborating that the downregulation of the GFP gene operates via RNAi-mediated mechanism. According to the obtained data, polyplexes formed at P/N/C ratios of 1:16:8 and 1:16:16 were found to be effective in GFP gene silencing. The most pronounced effect was observed with siRNA/R6p/CDP-E6Hp polyplexes formed at a 1:16:16 ratio. It is worth noting that a significant difference (*p* < 0.05) was found between CDP-ligand-modified and control polyplexes formed at this charge ratio. Thus, it can be assumed that the ligand modification led to an increase in transfection efficiency. Earlier, we showed that the inclusion of a CDP-ligand sequence can improve the transfection efficiency and specificity of cationic peptide-based DNA and siRNA delivery systems [11,28,56]. However, in this study, we successfully demonstrated that a similar effect can be achieved by modifying an anionic peptide-based coating.

### 2.6. Evaluation of the Trasnfection Properties of Ternary Polyplexes In Vivo

Rat model of EM was surgically induced by subcutaneous autotransplantation of uterine horn fragments as described previously Rats with a regular estrous cycle were included to the experiment, no animals were excluded in the current study [11,48,57,58]. Two tissue fragments were autotransplanted onto the external surface of the abdominal wall in each rat (Figure S2). The left implant was injected twice, with a one-week interval between injections, receiving a total siRNA dose of 10 µg. Control animals were injected with saline. The right implants remained untreated. Animal body weights were monitored throughout the experiment, and no significant variations were observed. The transfection properties of the polyplexes and their therapeutic potential were assessed by measuring the volume of endometrial implants, determining *VEGFA* gene expression in the implants, and performing immunohistochemical analysis of the vascular marker CD34 on implant sections.

**Figure 5:**
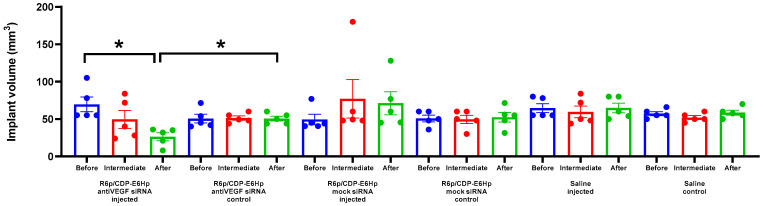
Volume of endometriotic implants in rats measured before and after the treatment with the anti-VEGFA or mock siRNA/R6p/CDP-E6Hp polyplexes. *—*p* < 0.05 when compared with animal before treatment and treated with mock siRNA. The data are shown as the mean ± S.E.M. of n = 5 individual samples; statistical significance was assessed by Kruskal–Wallis test with Dunn’s correction.

Measurements of the implants volume was performed three times: prior to the initial injection, prior to the subsequent injection, and at the final stage of experiment. Results of the measurements are illustrated in Figure 5.

The data presented indicate that a significant 2.6-fold reduction in implant size was observed exclusively after the administration of anti-VEGFA siRNA. As illustrated in Figure 5, no reduction in implant size was detected following the injection of the corresponding control siRNA/R6p/CDP-E6Hp polyplexes. Consistent with expectations, no reduction in volume was observed in the control contralateral implants. A statistically significant difference in volume was identified between the injected implants and the intact implants (Figure 5).

**Figure 6:**
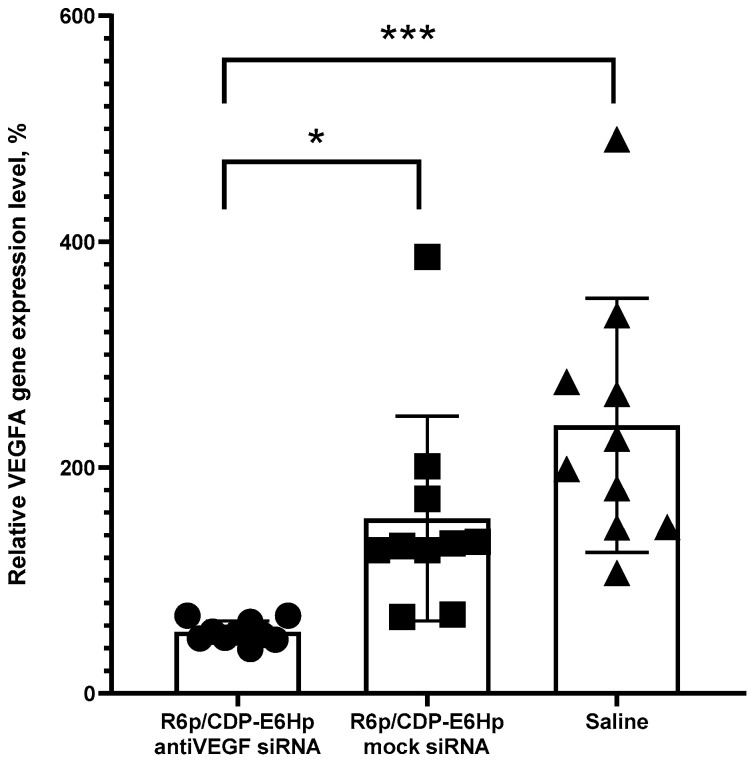
In vivo silencing of *VEGFA* gene expression in endometrial implants following administration of anti-VEGFA or control siRNA/R6p/CDP-E6Hp polyplexes. Expression levels are presented relative to those observed in untreated implants. *—*p* < 0.05 when compared with mock siRNA-polyplexes; ***—*p* < 0.001 when compared with saline. The data are shown as the mean ± S.D. of n = 5 animals per experimental group and two independent measurements per animal; statistical significance was assessed by ordinary one-way ANOVA.

Molecular genetic analysis of *VEGFA* gene expression was conducted on both injected and contralateral implants. Gene expression levels in intact implants were assumed to represent the baseline (100%) (Figure 6).

The data presented in Figure 6 demonstrate a marked downregulation of *VEGFA* expression exclusively following administration of ternary polyplexes containing specific siRNA, whereas injections with mock siRNA did not elicit such an effect. This result confirms the RNAi-mediated mechanism underlying target gene silencing. Relative to the expression levels observed in the intact contralateral implants, treatment resulted in an approximate twofold reduction in *VEGFA* expression. Notably, injections of mock siRNA polyplexes and saline induced a significant upregulation of the target gene, likely attributable to injection-induced tissue trauma. Despite this, the level of *VEGFA* gene silencing observed after anti-VEGFA siRNA polyplexes administration was even more pronounced, exhibiting a 2.8-fold decrease compared to mock siRNA polyplexes injection and a 4.3-fold decrease relative to saline injection.

**Figure 7:**
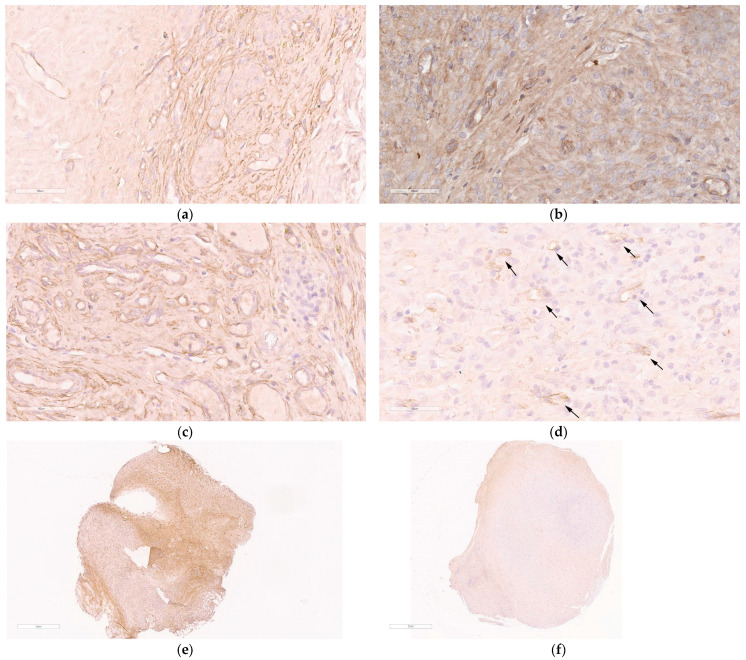
The immunohistochemical analysis of CD34-immunostained paraffin-embedded sections of endometriotic implants: (**a**) intact contralateral implant; (**b**) implant after the treatment with the mock siRNA/R6p/CDP-E6Hp polyplexes; (**c**) implant after injection of saline; (**d**) implant after the treatment with the anti-VEGFA siRNA/R6p/CDP-E6Hp polyplexes, arrows indicate areas of VEGFA expression (magnification 400×, bar represents 60 µm); (**e**) full-size image of intact contralateral implant; (**f**) full-size image of implant treated with the anti-VEGFA siRNA/R6p/CDP-E6Hp polyplexes (magnification 40×, bar represents 1 mm).

The anti-angiogenic effect of anti-VEGFA siRNA/R6p/CDP-E6Hp administration was assessed via immunocytochemical staining of CD34 antigen, which is expressed on hematopoietic progenitor cells and endothelial cells, making it a valuable marker for identifying endothelial cells [59]. Immunostaining for CD34 enables the detection of microvessel development, which plays a crucial role in supporting the growth of endometriotic implants. Due to limited amount of pathological tissue, we performed qualitative analysis of CD34 surface expression in endometriotic implants (Figure 7).

As illustrated in Figure 7a, an abundant surface expression of CD34 is present on the section of the intact implant. A similar pattern was observed after injection with mock siRNA/R6p/CDP-E6Hp polyplexes and saline (Figure 7b,c). In contrast, sections from implants treated with anti-VEGFA siRNA/R6p/CDP-E6Hp demonstrated a distinct pattern of CD34 expression. Specifically, these implants were characterized by a significantly reduced area of CD34 staining and a diminished number of microvessels, indicating a marked effect of the anti-VEGFA siRNA treatment (Figure 7d). A marked difference between intact and anti-VEGFA siRNA-treated implants is evident in Figure 7e,f, which show full-size views of immunostained sections. Extensive brown staining is observed in a section of the control implant, whereas CD34-specific staining is reduced in the treated implant (Figure 7e,f). Based on the obtained results, it can be supposed that the anti-VEGFA siRNA/R6p/CDP-E6Hp formulation can suppress microvessels formation in the treated implants.

## 3. Discussion

Nanoparticle-mediated gene therapy holds considerable promise for transforming the treatment landscape of female-specific diseases [60]. Nanoparticles serve as an innovative drug delivery system applicable to a range of gynecological conditions, including uterine leiomyoma, endometriosis, polycystic ovarian syndrome, and various gynecological cancers [61]. Among the diverse delivery platforms, peptide-based nanoparticles are particularly advantageous due to their versatility, biodegradability, and a modular carrier design. Incorporating different modules to overcome barriers to nucleic acid transport can significantly improve the efficiency and specificity of delivery [46,62].

In this study, we report the in vitro and in vivo characterization of peptide-based polyplexes featuring an anionic coating, designed as vehicles for the delivery of anti-VEGFA siRNA aimed at treating EM in a rat model. To optimize siRNA delivery to endometrial implants, the polyplexes were functionalized with a CXCL12-derived ligand targeting the CXCR4 chemokine receptor. Prior research has established that the CXCL12/CXCR4 signaling axis is hyperactivated in EM [22,24,63]. The upregulation of CXCL12/CXCR4 is implicated in the recruitment and homing of circulating stem cells to EM lesions, potentially inhibiting or modulating physiological uterine angiogenesis [64]. Furthermore, local expression of CXCR4 is critical for the proliferation of the epithelial compartment within EM lesions and may facilitate immune evasion mechanisms [65]. Previously, we demonstrated that CXCR4 can be used to target cationic peptide-based nanoparticles for efficient anti-VEGFA siRNA delivery both in vitro and in vivo [11,28]. To enhance serum stability and mitigate cytotoxicity associated with high cationic charge density, the current study employed an anionic glutamate-histidine-rich coating and included physicochemical characterization of the resulting polyplexes.

Zeta-potential measurements confirmed a substantial reduction in cationic charge; nonetheless, the polyplexes maintained stability, with no significant siRNA release detected by the SybrGreen exclusion assay (Figure 1 and Figure 2). Although nucleic acid nanoparticles with near-neutral surface charge typically exhibit aggregation, which can adversely affect transfection efficiency, several studies have elucidated mechanisms by which large polyplexes achieve effective nucleic acid delivery. These include disaggregation during the transfection process and uptake via macropinocytosis [66,67,68].

The clinical translation of non-viral vectors can be hindered by the significant cytotoxicity of polyplexes, which arises from multiple mechanisms, with high charge density playing a major role. Highly charged polyplexes can disrupt the integrity of the lipid bilayer, bind to serum proteins leading to the formation of large aggregates, or trigger the complement system [53]. We assessed the toxic properties of anionically coated polyplexes and found no cytotoxicity in any of the tested formulations (Figure 3). This represents a marked difference from our previous results obtained with cationic siRNA/R6p polyplexes, which exhibited 33.7% cytotoxicity at the same P/N ratio in MDA-MB-231 cells [48]. Indeed, many studies on ionic coating of polyplexes have reported a significant decrease in cytotoxicity levels, which can be considered an important advantage of these delivery systems [32,55].

Evaluation of RNAi efficiency following the delivery of anti-GFP or mock siRNA in CXCR4-positive breast cancer cells stably expressing *eGFP* gene revealed a marked difference between specific and control polyplexes. The absence of significant changes in GFP gene expression after treatment with mock siRNA in contrast to the pronounced silencing observed with anti-GFP siRNA delivery, unequivocally demonstrates an RNAi-based mechanism of gene silencing (Figure 4). It is essential to highlight that the transfection experiments were conducted in a serum-enriched medium, which is used to evaluate the serum resistance of delivery vectors before proceeding to in vivo studies because both serum proteins and components of the extracellular matrix can disassemble polyplexes [69]. The high cationic charge density of polyplexes can negatively impact their in vivo performance [70]. One effective strategy to mitigate this issue is the ionic coating of polyplexes with anionic polymers, such as hyaluronic acid and polyglutamic acid [32,40,71,72]. The significant silencing of *eGFP* expression in vitro, mediated by anti-GFP siRNA/R6p/CDP-E6Hp polyplexes at the optimal charge ratio, suggests that coating with anionic peptide-based polymers can facilitate successful siRNA transfection of cells under physiologically relevant conditions. This suggestion enabled us to conduct an in vivo assessment of the therapeutic potential of the developed siRNA delivery system in an animal model of EM.

For the in vivo evaluation of the therapeutic potential of the developed polyplexes, we used a rat model of EM established via subcutaneous autotransplantation of endometrial tissue. The subcutaneous localization of EM implants made them easily accessible for manipulation and allowed us to measure the surgically induced implants three times during the experiment. Consistent with the in vitro data, we observed a significant decrease in implant growth only after administration of anti-VEGFA-bearing polyplexes (Figure 5). However, this effect was detected only at the final measurement. It is worth noting that a single injection of anti-VEGFA siRNA/R6p/CDP-E6Hp polyplexes did not result in a significant reduction in implant volume; only a trend toward reduction was observed. This suggests that a higher dose may be required for a rapid response or that more time is needed to achieve a therapeutic effect. Nevertheless, in this study, we demonstrate that the administration of anionic ternary polyplexes can reduce EM implant volume by 2.6-fold, which is comparable to the efficiency of previously studied nanoparticles carrying anti-angiogenic siRNAs [48,58,73,74]. Moreover, in those studies, we observed an approximately 2- to 2.5-fold reduction in implant volume following pharmacotherapy with Dienogest, a leading drug for the treatment of EM. Therefore, the results of the current study can be favorably compared with previous data on EM treatment in a rat model [48,58,75].

Quantitative molecular analysis of transfection efficiency in vivo revealed a marked decrease in *VEGFA* gene expression in the experimental group but not in the control group (Figure 6). Specifically, a several-fold reduction in target gene expression was achieved, demonstrating the efficient and specific action of the developed formulation on EM implants. Furthermore, immunohistochemical analysis of CD34 surface expression on EM implant sections corroborated the molecular data, as we observed distinct patterns of specific immunostaining between the treated and control groups (Figure 7).

The findings presented in this study align with prior research on RNAi-based therapeutic strategies for EM. Several studies have demonstrated effective delivery of small siRNA and miRNA for EM treatment utilizing non-viral nanoparticles or exosomes, as comprehensively reviewed by Maestas-Olguin et al. [60]. Collectively, these results underscore the potential of RNA interference as a viable therapeutic modality for EM. Notably, the majority of delivery platforms that have achieved efficient systemic administration possess either an anionic or neutral surface charge, highlighting the importance of surface modification in overcoming extracellular barriers to the in vivo transport of nucleic acids [60].

## 4. Materials and Methods

### 4.1. Cell Lines and Animal Models

Human triple-negative breast cancer cells MDA-MB-231, stably expressing gene of green fluorescent protein, were cultured in the absence of mycoplasma contamination, adhering to previously established protocols [29].

This study utilized a cohort of fifteen female Wistar rats, each twelve weeks of age and non-pregnant. The subjects were procured from the Rappolovo Breeding Center (Saint-Petersburg, Russia) and exhibited body weights ranging from 180 to 250 g. The animals were maintained in a controlled laboratory environment with ad libitum access to water and a standard laboratory diet. A two-week acclimatization period was implemented prior to surgical procedures. Inclusion criteria for the experimental protocol required that the rats demonstrate a regular estrous cycle of 4 to 5 days in duration. The experimental surgical procedures were conducted in accordance with the principles outlined in the Helsinki Declaration. Additionally, the study protocol was reviewed and approved by the Ethics Committee of the D.O. Ott Research Institute of Obstetrics, Gynecology, and Reproductology.

### 4.2. Synthesis of Peptide Carriers

R6 (CHRRRRRRHC), E6H (CHHEEEEEEHHC), and CDP-E6H (KPVSLSYRSPSRFFESHXXCHHEEEEEEHHC) peptides were synthesized using solid phase Fmoc-chemistry at NPF Verta, LLC in Saint-Petersburg, Russia.

The peptides were preserved in a lyophilized form at a controlled temperature of −20 °C to maintain their stability. Their purity was accessed via high-performance liquid chromatography (HPLC), revealing purity levels between 90% and 95%. Furthermore, the peptide-based polymers R6p, E6Hp, and CDP-E6Hp employed in this study were synthesized following an established protocol [45]. In summary, the peptides were dissolved to a concentration of 30 mM in a solution containing 30% DMSO and subsequently underwent an oxidative polycondensation reaction for a duration of 96 h. The synthesized polymers were then preserved in aqueous solution at a concentration of 2 mg/mL and stored at −70 °C. The quantity of residual unreacted thiol groups was quantified utilizing Ellman’s assay and reported as a percentage relative to the absorbance values obtained from the unpolymerized peptide samples [45].

### 4.3. Sequences of siRNA

siRNAs were synthesized at Syntol JSC (Moscow, Russia). The anti-GFP siRNA, with the sense strand sequence of 5′-CAA GCU GAC CCU GAA GUU Ctt-3′, as well as the anti-VEGFA siRNA, with the sense strand sequence of 5′-GCG GAU CAA ACC UCA CCA Att-3′, were reported previously [29]. For experimental control, a mock siRNA with the sense strand sequence 5′-UUC UCC GAA CGU GUC ACG Utt-3′ was used [28].

### 4.4. Preparation of Ternary Polyplexes

Ternary polyplexes composed of siRNA, peptide, and coating were formulated using various P/N/C ratios, denoting the molar ratios of siRNA phosphorus to peptide nitrogen and coating carboxyl groups, respectively. Initially, siRNA/R6p complexes were formulated at a P/N ratio of 1:16 in Hepes-buffered mannitol (HBM) solution, which contained 5% (*w*/*v*) mannitol and 5 mM Hepes at pH 7.5, followed by thorough mixing [45]. Subsequently, the complexes were incubated at ambient temperature for 30 min. Following this, negatively charged E6Hp or CDP-E6Hp coatings were added to the complexes at varying P/C ratios and allowed to incubate for an additional 30 min to facilitate the formation of ternary polyplexes. As a control, the Turbofect transfection reagent (Thermo Fisher Scientific, Waltham, MA, USA) was employed in accordance with the manufacturer’s protocol.

### 4.5. SybrGreen Exclusion Assay

To assess the relaxation of polyplexes induced by anionic coating, we employed the SYBR Green displacement assay, as described previously [28]. Fluorescence quenching of SYBR Green was measured using the Wallac 1420D scanning multilabel counter (PerkinElmer Wallac Oy, Turku, Finland). Binding efficiency was determined according to the equation (F − Ff)/(Fb − Ff), where Ff and Fb denote the fluorescence intensities of SYBR Green in the absence and presence of siRNA, respectively. The fluorescence value corresponding to unbound siRNA was used as the 100% reference standard.

### 4.6. Measurement of Size and Zeta-Potential of Ternary Polyplexes

Ternary polyplexes were formulated following the protocol described above. Each formulation consisted of 5 µg of siRNA, with a range of P/N/C ratios evaluated. The particle size of the polyplexes was assessed via dynamic light scattering, and the zeta-potential was quantified using microelectrophoresis. All measurements were performed in triplicate employing a Zetasizer NANO ZS instrument (Malvern Instruments, Malvern, UK).

### 4.7. Cytotoxicity Evaluation of Ternary Polyplexes

The cytotoxic effects of ternary polyplexes were evaluated at a P/N ratio of 1:16 and across a range of P/C ratios from 1:4 to 1:24 in the MDA-MB-231 cell line. The assessment was performed in 96-well plates utilizing the Alamar Blue assay (BioSources International, San Diego, CA, USA). Cell viability was measured after 16 h of incubation, according to a previously established protocol [58]. After transfection the fluorescence of resorufin was measured using a Wallac 1420D scanning multilabel counter with excitation 544 nm and emission 590 nm wavelengths. The relative fluorescence intensity was calculated as (F − Ff)/(Fb − Ff) × 100%, where Fb and Ff represent the fluorescence intensities in the untreated control and without cells, respectively.

### 4.8. siRNA Transfection and GFP Fluorescence Detection

The siRNA transfection experiments were conducted in triplicate using MDA-MB-231 cells that stably express GFP [58]. A total of 70 × 10^4^ cells were seeded in 48-well plates and left to incubate overnight. The transfections took place in serum-supplemented medium without the medium changing. Complexes containing anti-GFP siRNA or mock siRNA at concentrations of 200 nM were formed at various P/N/C ratios and incubated with the cells for 48 h. After incubation the cells were washed with 1× PBS (pH 7.2) and made permeable using a cell lysis buffer containing 25 mM Gly-Gly, 15 mM MgSO_4_, 4 mM EGTA, 1 mM DTT, and 1 mM PMSF at pH 7.8. The GFP fluorescence was then measured using a Wallac 1420D counter with excitation and emission wavelengths of 485 and 535 nm, respectively. The fluorescence level was normalized by determining the protein concentration in each sample using the Bradford method.

### 4.9. Induction of EM in Rat Model and In Vivo siRNA Transfection

We conducted surgical modeling of EM in accordance with established protocols [11,57,58]. In this study, two autologous uterine tissue fragments were transplanted onto the external surface of the abdominal wall in ovariectomized rats. Prior to the transfection procedures, the implants were allowed a two-week growth period. A total of twenty-two rats were randomly assigned to three groups (*n* = 5 per group). Over the course of one week, two administrations of either anti-VEGFA siRNA or control (mock) siRNA, totaling 10 µg, were delivered in the form of siRNA/R6p/CDP-E6Hp polyplexes at a P/N/C ratio of 1:16:16, with a one-week interval between injections. Under anesthesia, one endometrial implant in each rat received siRNA-complex injections at a dose of 5 µg, while the contralateral implant remained untreated. In the negative control group, endometrial implants were injected with an equivalent volume of saline. One week following the initial injection, the procedure was repeated, and the animals were euthanized one week thereafter. Volumetric measurements of both the injected and contralateral implants (calculated as length × width × 2 mm^3^) were recorded prior to the first injection, before the second injection, and after the treatment period. Subsequent analyses included immunohistochemical detection of CD34 and assessment of VEGFA gene expression, conducted according to previously established protocols Experimental samples at this stage of the analysis were blinded [58]. In summary, tissue sections measuring 3–4 µm in thickness were stained utilizing the BOND-MAX immunohistostainer (Leica Biosystems, Nussloch, Germany) with mouse monoclonal primary antibody targeting CD34 (clone EP373Y, Abcam, UK). Subsequently, images (n = 4 per implant) were captured employing a Leica Aperio AT2 slide scanner and analyzed using Aperio ImageScope software v.6.25. To assess VEGFA and β-actin gene expression in EM implants of VEGFA, we employed quantitative real-time PCR with previously reported primers [29]. Each sample was divided and measured separately. Similarly, we used a comparable protocol to assess VEGFA gene expression in vivo in EM implants. We compared the expression level of VEGFA gene in vivo to the expression level in control animals injected with saline.

### 4.10. Statistical Analysis

Statistical analyses were conducted using the GraphPad Prism version 8 software (GraphPad Prism Inc., San Diego, CA, USA). Statistical significance was defined as * *p* < 0.05, ** *p* < 0.01, and *** *p* < 0.001.

## Figures and Tables

**Table 1:** The formulas of monomers and carriers. X — ε-aminocaproic acid.

	Name	Composition
Monomers	R6	CHRRRRRRHC
	E6H	CHHEEEEEEHHC
Ligand monomer	CDP-E6H	KPVSLSYRSPSRFFESHXXCHHEEEEEEHHC
Polycondensed carriers	R6p	(CHRRRRRRHC)n
	E6Hp	(CHHEEEEEEHHC)n
	CDP-E6Hp	(KPVSLSYRSPSRFFESHXXCHHEEEEEEHHC)n

## Data Availability

The original contributions presented in this study are included in the article/Supplementary Material. Further inquiries can be directed to the corresponding author.

## Supplementary Materials

The following supporting information can be downloaded at: https://www.mdpi.com/article/10.3390/ijms262110582/s1.
