# Supplementary material for: Anti-Angiogenic RNAi-Based Treatment of Endometriosis in a Rat Model Using CXCR4-Targeted Peptide Nanoparticles
Source: Int J Mol Sci. 2025 Oct 30;26(21):10582. doi: 10.3390/ijms262110582 (PMC12607363; doi:10.3390/ijms262110582)
Supplement: Supplementary file 1 [file ijms-26-10582-s001.zip › ijms-3935916-supplementary.pdf]

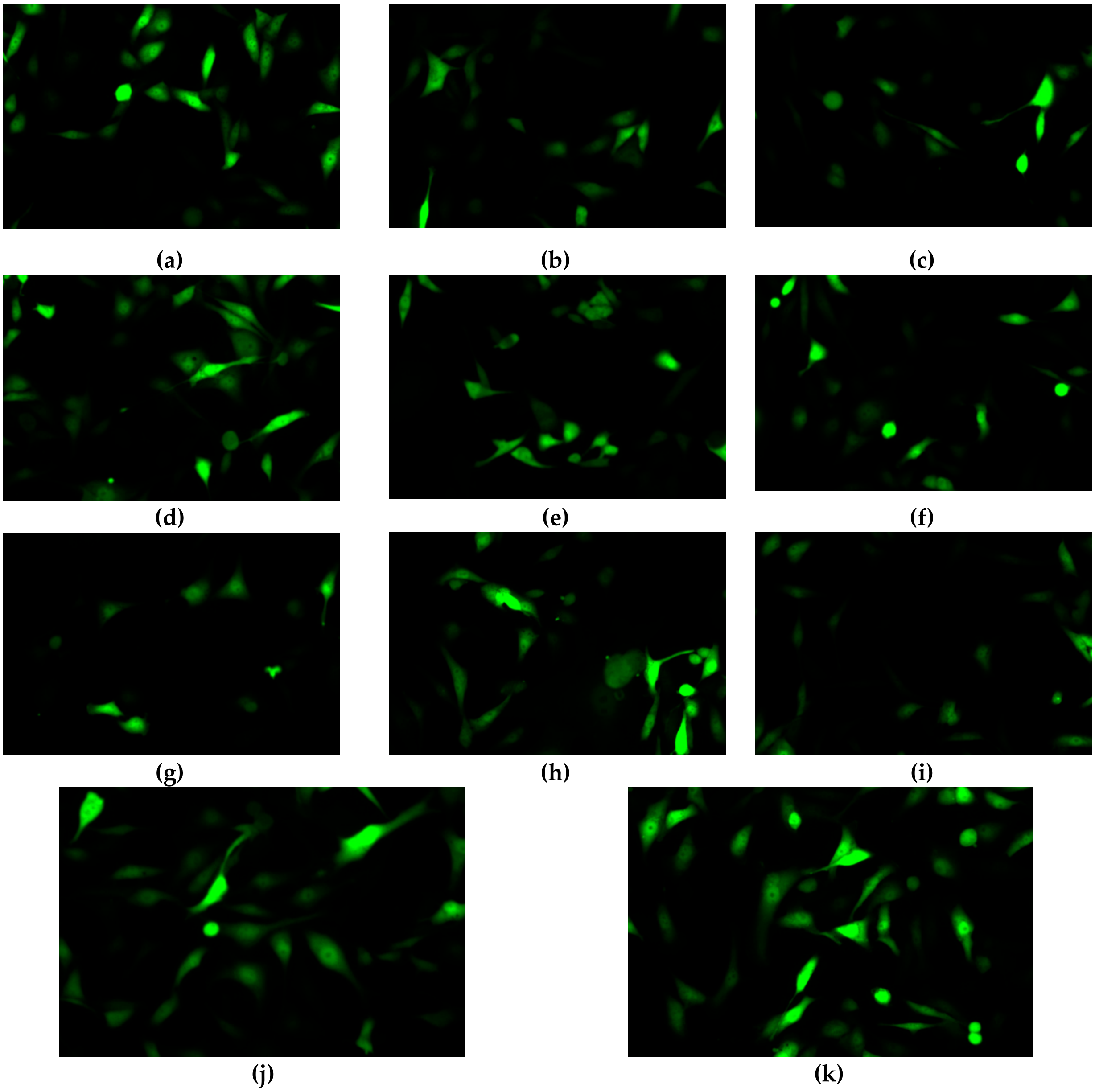

Figure S1. Representative micrographs of MDA-MB-231 cells stably expressing GFP gene treated by the ternary polyplexes formed with anti-GFP siRNA, R6p carrier and E6Hp **(a-d)** or CDP-E6Hp coating **(e-h)** at different P/N/C ratios: **(a, e)** 1/16/4; **(b, f)** 1/16/8; **(c, g)** 1/16/16; **(d, h)** 1/16/24. Positive control: **(i)** MDA-MB-231 cells treated by anti-GFP siRNA/Turbofect. Negative controls: **(j)** MDA-MB-231 cells treated by mock siRNA/Turbofect and **(k)** intact cells. Micrographs of GFP-positive cells were recorded using a Leica DM 2500 microscope (Wetzlar, Germany) at ×200 magnification.

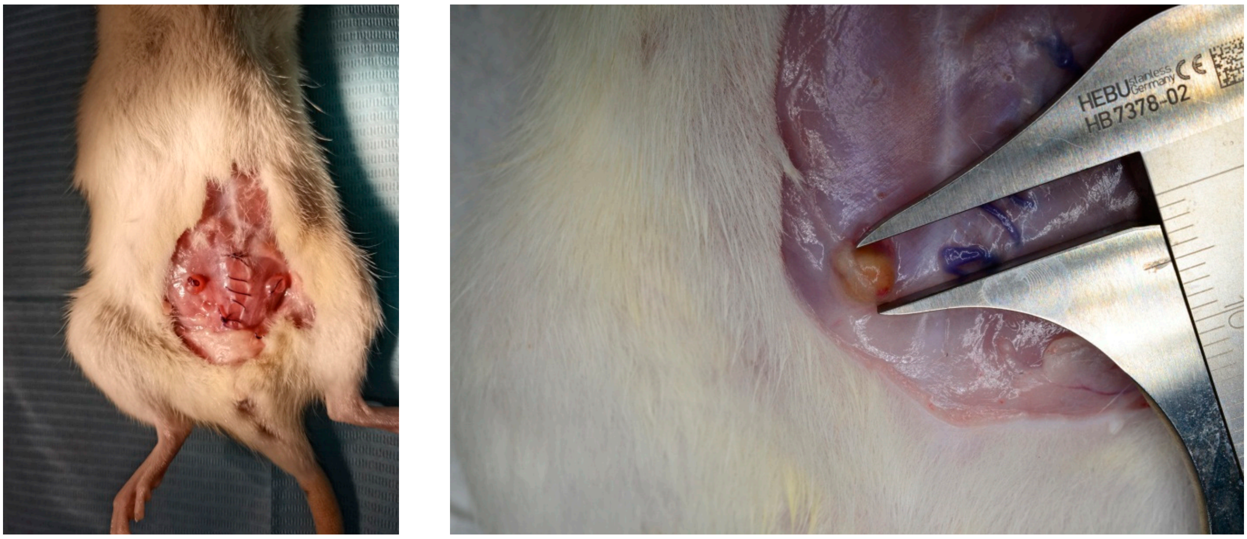

(a) **(b)**

Figure S2. Typical appearance of EM implants after autopsy: **(a)** control EM implants; left implant was injected with saline; **(b)** measurement of the implant size after autopsy.
